# Myopéricardite aiguë simulant un infarctus du myocarde: à propos d'une observation et revue de la littérature

**DOI:** 10.11604/pamj.2015.21.70.6530

**Published:** 2015-05-28

**Authors:** Abdelmajid Bouzerda

**Affiliations:** 1 Unité d'Hémodynamique et de Cardiologie Interventionnelle, Centre Médico-chirurgical, Agadir, Maroc

**Keywords:** Myopéricardite, syndrome coronarien aigu, IRM cardiaque, myopericarditis, acute coronary syndrome, cardiac MRI

## Abstract

Le diagnostic de myopéricardite aiguë est difficile surtout quand la présentation clinique mime un syndrome coronaire aiguë. Nous rapportons l'observation d'un patient âgé de 40 ans admis pour un syndrome coronarien aiguë ST+ chez qui la coronarographie réalisée en urgence montre un réseau coronaire angio-graphiquement sain. L'imagerie par résonance magnétique (IRM) permettra d'affirmer le diagnostic d'une myopéricardite. Cette observation confirme le rôle primordial de l'IRM cardiaque chez les patients présentant une suspicion clinique de myocardite aiguë ou un tableau de SCA à coronaire saines.

## Introduction

La myocardite peut revêtir des tableaux cliniques très divers. Si les formes avec insuffisance cardiaque congestive, troubles du rythme ou cardiopathies dilatées sont fréquentes, les formes pseudo nécrotiques sont plus rares et surtout plus trompeuses. Le diagnostic de myocardite aiguë (MA) est difficile. Il est basé sur l'association d'arguments cliniques, électriques, biologiques et morphologiques et constitue souvent un challenge pour le praticien [1]. Nous rapportons l'observation d'une myocardite prise au préalable pour un syndrome coronarien aiguë.

## Patient et observation

**Figure 1:**
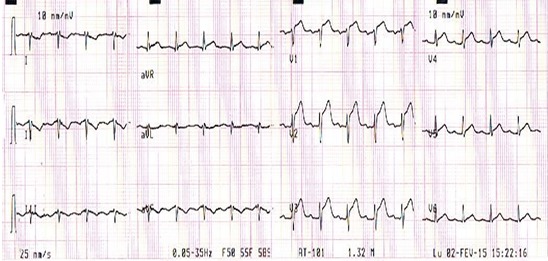
Sus décalage ST en antérieur

Monsieur E.A. âgé de 40 ans, est admis aux urgences pour une douleur thoracique constrictive évoluant depuis 24 h, au décours d'un syndrome grippal. Ce patient est non fumeur, sans facteur de risque vasculaire connu ni antécédent notable. L'examen clinique d'admission trouve un état hémodynamique stable avec une Tension artérielle à 135 / 85 mmhg et une fréquence cardiaque à 75 battements par minute. A l'auscultation cardiaque le rythme est régulier, sans souffle ni frottement. les poumons sont secs à l'auscultation sans signe périphérique d'insuffisance cardiaque et le reste de l'examen somatique est sans particularité. L’électrocardiogramme basal de repos inscrit un sus-décalage du segment ST en antérieur (Figure 1). La radiographie de thorax est normale. L’échocardiographie retrouve un VG de taille normale, une hypocinésie de la paroi inféroseptale, une fonction systolique ventriculaire gauche conservée (FE à 70% par le simpson biplan), un péricarde libre sans valvulopathie mitroaortique.

**Figure 2:**
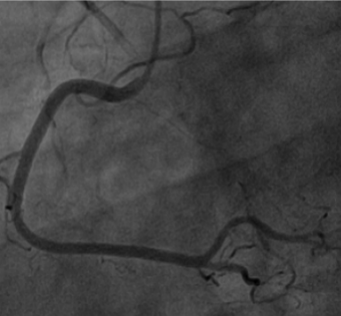
Aspect angiographique normal de l'artère Coronaire droite

**Figure 3:**
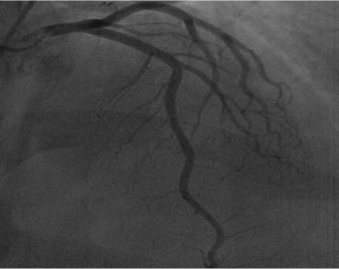
Aspect angiographique normal de l'artère inter-ventriculaire antérieure et de l'artère circonflexe

Au plan biologique: VS accélérée à 80 mm à la 1e heure; taux de fibrinogène élevé à 6,5 g/l; CRP à 150 mg /l; CPK élevées à 395 UI/l avec une fraction MB à 55.4 UI/l troponine i positive à 7,93 ug/l. Un traitement pharmacologique à base de Clopidogrel (Plavix) 600 mg, Enoxaparine (Lovenox) IV 0.5mg /kg, Aspégic 250 mg IV, Morphine en Sc est instauré, puis adressé en urgence pour coronarographie. Celle-ci a visualisé un réseau coronaire angio-graphiquement sain (Figure 2, Figure 3). L’évolution clinique a été simple sur le plan hémodynamique et rythmique sans récurrence douloureuse.

**Figure 4:**
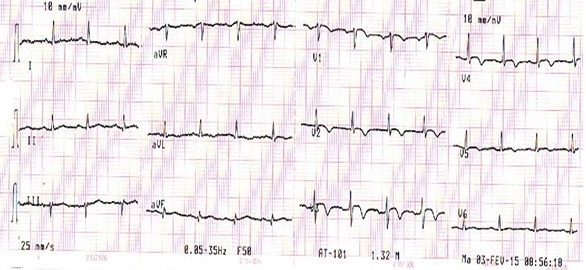
Ondes T négatives en antérieures

**Figure 5:**
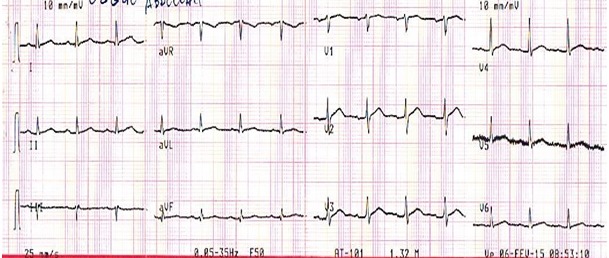
Normalisation des ondes T en antérieures

Sur le plan électrique l’évolution est marquée par une négativation des ondes T en antérieur à J2 (Figure 4) puis une normalisation de l'ECG à J 4 (Figure 5). Les sérologies virales et bactériennes sont négatives. Le diagnostic de myopéricardite sèche est suspecté devant la normalisation du bilan inflammatoire sous traitement anti-inflammatoire d'autant plus que l'interrogatoire orienté a retrouvé les jours précédant l'hospitalisation du patient un syndrome pseudo-grippal. L'IRM cardiaque renforce ce diagnostique en montrant un aspect d'hypérsignal T2 et un rehaussement tardif intra-myocardique nodulaire de la paroi antéro-septale.

## Discussion

La présentation clinique de la myocardite aiguë est extrêmement variable, allant de la douleur thoracique ou dyspnée modérée au choc cardiogénique et parfois même au décès. La prise en charge diagnostique des patients et leur traitement sont fonction de la cause de la myocardite et restent peu ou non codifiés. L'incidence véritable de cette affection est délicate à déterminer compte tenu de l'existence de formes pauci ou asymptomatiques pouvant passer totalement inaperçues au cours d'une symptomatologie virale banale [1]. Les principales étiologies des myocardites sont infectieuses (virales, bactériennes, fungiques, parasitaires), toxiques (cocaïne, émétine, catécholamines, anthracyclines, radiothérapie), liées à une maladie de système (lupus, polymyosites, sclérodermie, sarcoïdose, panartérite noueuse), au peri-partum ou secondaires à un syndrome hyperéosinophilique.

Sur le plan clinique Une myocardite peut être aussi bien paucisymptomatique qu’être révélée par une mort subite récupérée en passant par des troubles du rythme ventriculaires ou supra-venticulaires, des troubles conductifs, une dyspnée ou une douleur thoracique mimant parfois un syndrome coronaire aigu (le cas de notre observation). Les prodromes couramment rencontrés sont ceux d'un syndrome pseudogrippal avec asthénie, fébricule, myalgies, toux, troubles intestinaux. Sur le plan électrique. Un ECG normal n’élimine pas le diagnostic. Les anomalies rencontrées sont aspécifiques: tachycardie sinusale, troubles de repolarisation (onde T ou segment ST). L'ECG peut mimer un syndrome coronaire aigu ou présenter les caractéristiques d'une péricardite (sus-décalage de ST diffus concave vers le haut, sous-décalage du segment PQ). Des troubles conductifs (Bloc auriculo-ventriculaire, Bloc de branche gauche de haut degré) sont plus fréquemment décrites lorsque l'histoire clinique est plus ancienne [2]. L’élévation de la troponine à la phase aiguë est plus fréquente que celle des CPK et peut être retrouvée tout au long du premier mois suivant l’épisode aiguë. La réalisation systématique d'examens virologiques ou bactériologiques a une très faible rentabilité. Seule la sérologie VIH doit être réellement proposée à chaque patient et il vaudra mieux ensuite privilégier un contexte clinique qui orientera éventuellement la recherche. L’échocardiographie permet de distinguer les myocardites aiguës et fulminantes [3]. Les myocardites fulminantes se caractérisent par une fonction ventriculaire gauche altérée, un épaississement du septum interventriculaire et par l'absence de dilatation ventriculaire gauche. Les myocardites aiguës montrent outre une altération de la fonction ventriculaire gauche, une dilatation du ventricule gauche et un septum interventriculaire de taille normale. L’échocardiographie permet également de diagnostiquer les myocardites focales. L'exploration coronaire doit être si ce n'est systématique, largement discutée. La coronarographie sert surtout à éliminer une cardiopathie associée, en particulier ischémique principal diagnostic différentiel devant un tableau clinique, biologique et échographique souvent peu spécifique.

L'IRM cardiaque élément clef du diagnostic permet d'affirmer le diagnostic de myocardite aiguë en objectivant des lésions de type inflammatoire, fixant le gadolinium au temps tardif et dont la topographie est, sous-épicardique. Les séquences morphologiques pondérées T2 permettent de visualiser des segments oedémateux qui apparaissent en hypersignal au sein du myocarde [4]. Les séquences dynamiques fournissent des arguments concernant la fonction systolique du VG (fraction d’éjection), la cinétique segmentaire, les mesures de diamètres, d’épaisseurs et les volumes des cavités cardiaques. Les séquences après injection de chélate de gadolinium, évaluent les dommages myocardiques représentés par un réhaussement tardif (RT) parfois nodulaire, de topographie sous-épicardique ou médioventriculaire mais non sous endocardique (ce qui le distingue des processus ischémiques) [4]. l'IRM permet un suivi précis des patients à distance de l’épisode aigu. Dans plus de 50% des cas, l’évolution se fait vers la guérison et les anomalies en IRM disparaissent à trois mois parallèlement à la normalisation de l'ECG et de l'ETT [5]. Par contre, certains patients conservent un hypersignal T2 et/ou un RT, y compris en l'absence de trouble de la cinétique segmentaire du VG, et alors que le bilan clinique et para-clinique est par ailleurs normalisé. On redoute alors un passage vers la chronicité et l’éventuelle évolution ultérieure vers une Cardiomyopathie dilatée qui peut comporter en IRM un aspect de RT analogue à celui rencontré au cours de la myocardite aigue [4, 6]. La réalisation d'une Biopsie endomyocardique conformément aux critères histologiques de Dallas, permet théoriquement d'affirmer le diagnostic [7]. Mais cet examen invasif n'est pas dénué de risques et souffre d'une très faible sensibilité (10 à 20%), les lésions étant souvent focales et nécessitant de multiples prélèvements, son interprétation nécessite une certaine expérience de cette pathologie sous peine d'une grande variabilité inter-observateurs [7].

Le traitement est fonction du tableau clinique. Compte tenu du risque hémodynamique mais aussi rythmique, toutes les myocardites et les myopéricardites doivent donc être hospitalisées à la phase aiguë pour une surveillance rapprochée. Dans la forme focale d'allure infarctoïde La prise en charge comporte le traitement de la douleur par des analgésiques, mais en étant relativement économe en matière d'anti-inflammatoires non stéroïdiens et même d'aspirine, qui seraient susceptibles, selon certaines données expérimentales, d'aggraver les dégâts myocardiques. Lorsqu'il existe une altération même modérée de la fonction ventriculaire gauche, les IEC sont indiqués. Les bêtabloquants trouvent leur indication lorsque l'atteinte myocardique se complique d'une hyperexcitabilité ventriculaire. Ces patients nécessitent de surcroît une surveillance à court et moyen termes de la fonction ventriculaire gauche. Les données de la littérature restent assez incertaines en matière d'histoire naturelle de ces myocardites aiguës d'allure initialement bénignes; il est très vraisemblable que la grande majorité d'entre elles guérissent sans séquelles; certaines détériorations retardées de la fonction ventriculaire gauche semblent néanmoins possibles, méritant donc une surveillance (échographique et ou IRM) renouvelée à échéance par exemple de 3 mois, 6 mois puis annuelle pendant 2 ou 3 ans [8].

Dans la forme diffuse et sévère le traitement est celui de l'insuffisance cardiaque aiguë et de ses éventuelles complications rythmiques, les indications dans les formes les plus graves d'une assistance circulatoire temporaire sont larges; il s'agit souvent de patients jeunes sans comorbidité et dont le pronostic à moyen terme peut être excellent, l’évolution vers une récupération quasi complète de la fonction ventriculaire une fois passée la période inflammatoire étant très probable.

## Conclusion

L'incidence réelle des myocardites est probablement sous-estimée du fait de la difficulté de la grande variété des formes cliniques et des difficultés diagnostiques liées à l'absence de méthode de référence aisément réalisable. Cette atteinte inflammatoire aiguë du myocarde a suscité récemment un net regain d'intérêt car deux nouveaux instruments diagnostiques ont permis de mieux la détecter et de mieux l'affirmer. Il s'agit, d'une part, de la généralisation des dosages de troponine permettant de détecter des dégâts cellulaires myocardiques a minima et, d'autre part, de l'IRM, permettant, par une analyse in vivo relativement précise de la structure myocardique, d'en établir le diagnostic.
